# Dedifferentiation: the return road to repair the intestinal epithelium

**DOI:** 10.1186/s13619-020-00048-2

**Published:** 2020-06-02

**Authors:** Yuan Liu, Xiaochen Xiong, Ye-Guang Chen

**Affiliations:** 1 The State Key Laboratory of Membrane Biology, Tsinghua-Peking Center for Life Sciences, School of Life Sciences, Tsinghua University, Beijing, 100084 China; 2 Institute of Stem Cell Biology & Regenerative Medicine, School of Medicine, Stanford University, Stanford, CA 94305 USA

## Abstract

In the March 5 issue of *Cell Stem Cell*, (Murata K et al. Cell Stem Cell. 26(377–390):e376 2020) reported that intestinal stem cell recovery after injury is principally through Ascl2-dependent dedifferentiation of absorptive and secretory precursors in mice. This study provides evidence for robust regenerative capability of the intestinal epithelium via dedifferentiation of absorptive and secretory progenitors in the crypt.

## Main Text

Many tissues in adults contain a population of tissue-specific stem cells, which are required for tissue homeostasis and repair (Blanpain and Fuchs, 2014; Li and Clevers, 2010). Most cells in the intestinal epithelium renew every 5 days. This quick renewal process is driven by the crypt base-resident Lgr5-marked intestinal stem cells (ISCs) (Barker, 2014). However, the intestinal epithelium harbors an extraordinary regenerative capacity to deal with repeatedly challenges, like various injuries or inflammation, to regenerate all cell-types and restore its normal architecture even without Lgr5^+^ ISCs.

Two models have been proposed to explain regeneration of the intestinal epithelium after damage (Barker et al., 2012). In the first model, known as “reserve stem cell” model, the quiescent reserve stem cells (also called + 4 cells), marked by Bmi1, Hopx, mTert and Lrig1, are regarded to be the regenerative cell type that can survive upon damage and repopulate Lgr5^+^ ISCs. The major challenge in this model is that expression of these markers traditionally seen as the quiescent stem cells is often not restricted to a given cell type, and whether the quiescent + 4 stem cells exist is still under debate (Barker et al., 2012). Recently, the other model has gained wide attention. In the “dedifferentiation” model, various cell types may serve as a source to dedifferentiate back to intestinal stem cells after epithelium damage (Bankaitis et al., 2018), which is accordance with the high plasticity of the intestinal epithelium (de Sousa and de Sauvage, 2019).

**Fig. 1:**
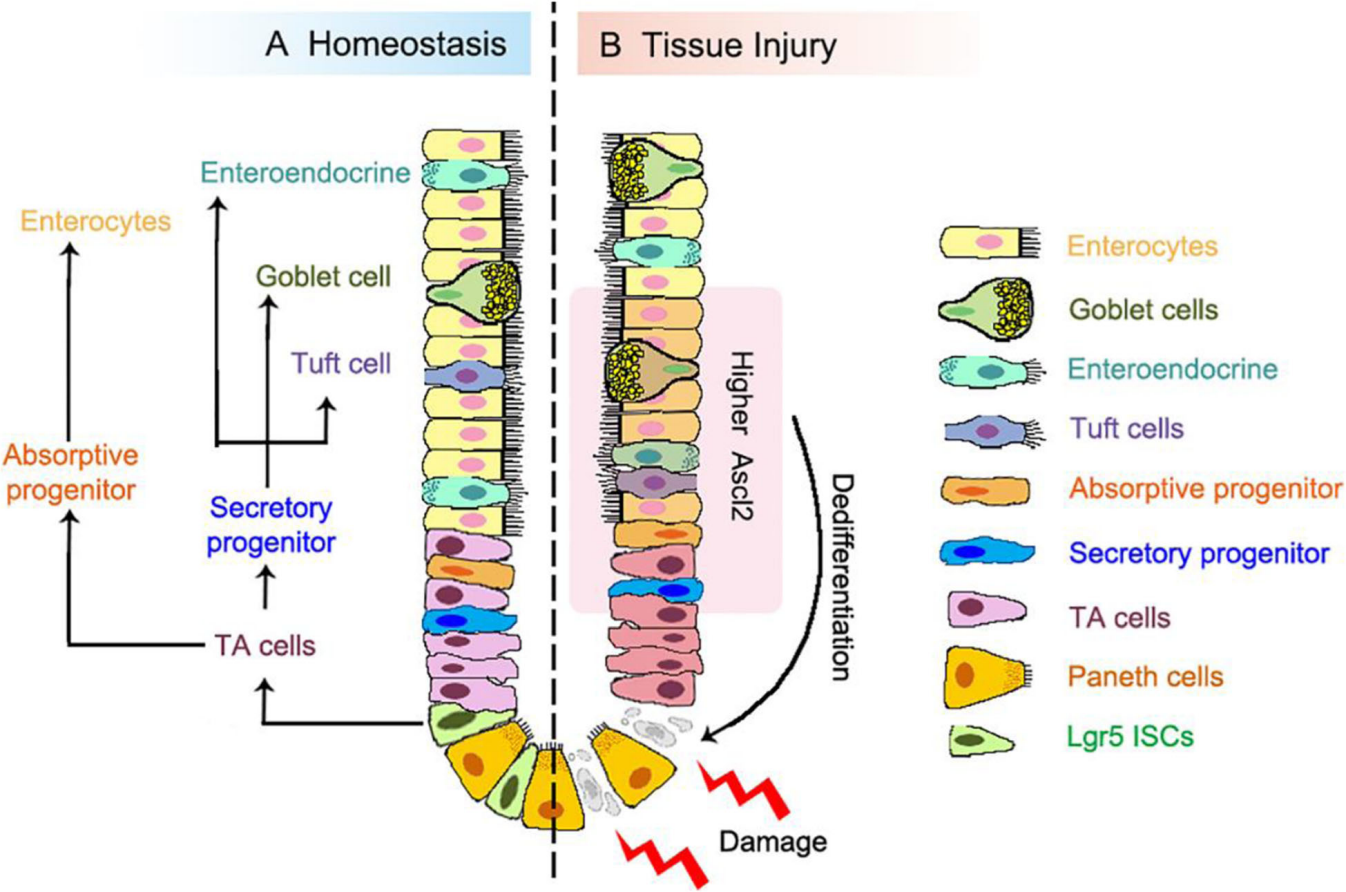
Intestinal epithelium during homeostasis and regeneration. **a** Homeostatic renewal of the intestinal epithelium is maintained by the actively cycling crypt base-resident Lgr5^+^ ISCs. These cells first generate transit amplifying (TA) cells, which continue moving up and differentiate into all cell types. **b** Tissue injury results in the loss of Lgr5^+^ cells and triggers the regeneration process to repair the epithelium. The differentiating progenitor cells in the crypt middle region gain Ascl2 expression, dedifferentiate and move to the base niche where they revert back to the Lgr5^+^ stem cell state

The transcription factor Ascl2 has been shown to play an important role in Lgr5^+^ ISC maintenance, and its expression is restricted to these stem cells (van der Flier et al., 2009). However, in the recent work, Murata et al. found that Ascl2 is largely dispensable for normal intestinal homeostasis, while Ascl2-deficiency impairs the refill of the Lgr5^+^ ISCs pool in both small intestinal and colonic regions after irradiation or ablation of Lgr5^+^ ISCs (Murata et al., 2020). These Ascl2-null mice display much shorter lifespan upon the lethal irradiation treatment. Upon the Diphtheria toxin-mediated Lgr5^+^ ISC depletion, the mCherry-marked Ascl2^+^ stem cells, which normally locate in the bottom of the crypt, disappear in the first few days and then re-appear in the middle region of colonic crypts at day 8. These cells move down into the bottom of the crypt at day 10, where they eventually express Lgr5 and regain stemness (Fig. 1). These results suggest that ISCs ablation-activated Ascl2 expression in the cells of the middle crypt region drives these cells to de-differentiate and repopulate the stem cell pool in the bottom region of the crypt. Supporting this, RNA-seq analysis revealed that regenerating Ascl2^+^ cells have a gene expression signature associated with absorptive cells or secretory cells and exhibit ISC-oriented dedifferentiation, and pseudotime analysis indicates a transition from the absorptive or secretory state to dedifferentiating state, and eventually reach the endpoint that acquire ISC characteristics. The authors further exploited the Ascl2 targets that mediate dedifferentiation. Among the upregulated genes in the middle region Ascl2^+^ cells, the interleukin-11 (IL-11) receptor Il11ra1 is a functional target of Ascl2 during regeneration. Consistently, recombinant IL-11 augments the organoids formation of the middle region Ascl2^+^ cells but not resting ISCs.

Their work prompts additional questions. Firstly, the important question is how epithelium damage or ISCs ablation triggers the dedifferentiation process. In another word, how do precursor cells sense the injury and initiate dedifferentiation? Extracellular matrix remodeling and YAP/TAZ signaling activation have been shown to be involved in the colonic regeneration in the dextran sulfate sodium (DSS) colitis mouse model (Yui et al., 2018), while no YAP/TAZ signaling signature is enriched in regenerating cells in the Murata et al.’s study. Secondly, single-cell RNA sequencing analysis revealed that the upper regenerative Ascl2^+^ cells comprise both absorptive and goblet progenitors. Do these two types of cells have an equal ability to revert to stem cells? Thirdly, there are two subpopulations with different proliferative activity in the upper Ascl2^+^ cells. Does there exist relationship between the actively cycling and regeneration? Fourthly, it remains unclear how IL-11 induces the dedifferentiation process. Fifthly, the complexity and redundancy of signals supporting ISC regeneration indicate the possibility that other target genes or signals may also contribute to the Ascl2-dependent dedifferentiation. Sixthly, this study demonstrated that Ascl2 re-expression in progenitors is critical for ISC repopulation after stem cell ablation or irradiation. However, it is unclear if this mechanism also applies to colitis-related epithelium repair. Moreover, after specific ablation of tumor Lgr5^+^ cells that have long-term self-renewal and differentiation capacities, differentiated KRT20^+^ cells can dedifferentiate into Lgr5^+^ cells (Shimokawa et al., 2017). Therefore, it will be interesting to compare signaling differences between physiological and tumor conditions. Addressing these questions will surely advance our understanding of tissue regeneration and provide solutions for regenerative medicine.

## References

[CR1] Bankaitis ED, Ha A, Kuo CJ, Magness ST (2018). Reserve stem cells in intestinal homeostasis and injury. Gastroenterology.

[CR2] Barker N (2014). Adult intestinal stem cells: critical drivers of epithelial homeostasis and regeneration. Nat Rev Mol Cell Biol.

[CR3] Barker N, van Oudenaarden A, Clevers H (2012). Identifying the stem cell of the intestinal crypt: strategies and pitfalls. Cell Stem Cell.

[CR4] Blanpain, C., and Fuchs, E. Stem cell plasticity. Plasticity of epithelial stem cells in tissue regeneration. Science 2018;344:1242281. 10.1126/science.1242281 PMC4523269 24926024

[CR5] de Sousa EMF, de Sauvage FJ (2019). Cellular plasticity in intestinal homeostasis and disease. Cell Stem Cell.

[CR6] Li L, Clevers H (2010). Coexistence of quiescent and active adult stem cells in mammals. Science.

[CR7] Murata K, Jadhav U, Madha S, van Es J, Dean J, Cavazza A, Wucherpfennig K, Michor F, Clevers H, Shivdasani RA (2020). Ascl2-dependent cell dedifferentiation drives regeneration of ablated intestinal stem cells. Cell Stem Cell.

[CR8] Shimokawa M, Ohta Y, Nishikori S, Matano M, Takano A, Fujii M, Date S, Sugimoto S, Kanai T, Sato T (2017). Visualization and targeting of LGR5(+) human colon cancer stem cells. Nature.

[CR9] van der Flier LG, van Gijn ME, Hatzis P, Kujala P, Haegebarth A, Stange DE, Begthel H, van den Born M, Guryev V, Oving I (2009). Transcription factor achaete scute-like 2 controls intestinal stem cell fate. Cell.

[CR10] Yui S, Azzolin L, Maimets M, Pedersen MT, Fordham RP, Hansen SL, Larsen HL, Guiu J, Alves MRP, Rundsten CF (2018). YAP/TAZ-dependent reprogramming of colonic epithelium links ECM remodeling to tissue regeneration. Cell Stem Cell.

